# Supplementary material for: Ex Vivo Treatment with Allogenic Mesenchymal Stem Cells of a Healthy Donor on Peripheral Blood Mononuclear Cells of Patients with Severe Alopecia Areata: Targeting Dysregulated T Cells and the Acquisition of Immunotolerance
Source: Int J Mol Sci. 2022 Oct 30;23(21):13228. doi: 10.3390/ijms232113228 (PMC9655710; doi:10.3390/ijms232113228)
Supplement: Supplementary file 1 [file ijms-23-13228-s001.zip › ijms-1978085-supplementary.pdf]

Table S1. PBMC in vitro real-time PCR primer sequences.

Species	Primer name	Forward (5′ – 3′)	Reverse (5′ – 3′)
Human	IL-1A	CAG TTC TGC TGA CTG GGT GA	AGG TGC TGA CCT AGG CTT GA
	IL-2RG	CCA CTC TGT GGA AGT GCT CA	TCC GTT CCA GCC AGA AAT AC
	IL-10	TCA TTC CCC AAC CAC TTC AT	GTA GAG ACG GGG TTT CAC CA
	IL-15	GCC TTC ATG GTA TTG GGA A	TGC TGT TAC TTT GCA ACT GG
	IL-17	ACC AAT CCC AAA AGG TCC TC	GGG GAC AGA GTT CAT GTG GT
	IL-18	AGC TGA AGA TGA TGA AAA CCT G	ATA GAG GCC GAT TTC CTT GG
	IFN-gamma	GCA TGG CTC TCC TCT TTC TC	CTG TGG CAT GAT CTG GTA CT
	CCR7	TGA GCG TGT CTT TGT TTT GC	CTT TCA GCC TCT TGG TCC TG
	CCR10	CTC AAT CCC GTT CTC TAC GC	GAT TCG CAG CCC TAG TTG TC
	Foxp3	TGC CTC CTC TTC TTC CTT GA	GAG CTG GTG CAT GAA ATG TG
	TNF-α	TCT ATC TGG GAG GGG TCT TC	TGA TCC CAA AGT AGA CCT GC
	TGF-β2	AAC TGT CTG CCC AGT TGT TA	GCT GAG ACG TCA AAT CGA AC
